# Outcomes of arthroscopic *"Remplissage"*: capsulotenodesis of the engaging large Hill-Sachs lesion

**DOI:** 10.1186/1749-799X-6-29

**Published:** 2011-06-15

**Authors:** Barak Haviv, Lee Mayo, Daniel Biggs

**Affiliations:** 1 Arthroscopy and Sports Injuries Unit, Hasharon Hospital, Rabin Medical Center, 7 Keren Kayemet St Petach-Tikva, 49372, Israel; 2 Central West Orthopedics and Sports Injuries, Suite 204, 30 Campbell St. Blacktown, NSW 2148, Australia; 3 Westmead Private Hospital, Cnr Mons & Darcy Roads, Westmead, NSW 2145, Australia

## Abstract

**Background:**

A Hill-Sachs lesion of the humeral head after a shoulder dislocation is clinically insignificant in most cases. However, a sizable defect will engage with the anterior rim of the glenoid and cause instability even after anterior glenoid reconstruction. The purpose of this study was to evaluate the outcome of arthroscopic capsulotenodesis of the posterior capsule and infraspinatus tendon ("remplissage") to seal a large engaging Hill-Sachs lesion in an unstable shoulder.

**Methods:**

This was a prospective follow-up study of patients who underwent arthroscopic surgery for recurrent shoulder instability with a large engaging Hill-Sachs lesion from 2007 to 2009. The clinical results were measured preoperatively and postoperatively with the Simple Shoulder test (SST) and the Rowe score for instability.

**Results:**

Eleven patients met the inclusion criteria of this study. The mean follow-up time was 30 months (range 24 to 35 months). At the last follow-up, significant improvement was observed in both scores with no recurrent dislocations. The mean SST improved from 6.6 to 11 (p < 0.001). The mean Rowe Score improved from 10.6 to 85 points (p < 0.001). On average patients regained more than 80% of shoulder external rotation.

**Conclusions:**

Arthroscopic *remplissage *for shoulder instability is an effective soft tissue technique to seal a large engaging Hill-Sachs lesion with respect to recurrence rate, range of motion and shoulder function.

## Introduction

Posterior-lateral compression fracture of the humeral head (a Hill-Sachs lesion) is a common finding associated with anterior shoulder instability [1-3]. Most Hill-Sachs lesions are clinically insignificant and do not require surgical treatment. However, Palmer and Widen [4] realized that a sizable defect will engage with the anterior rim of the glenoid and cause instability even after anterior glenoid reconstruction. The term engaging Hill-Sachs lesion was used by Burkhart and De Beer [5] to describe the leverage of the humeral head from the glenoid rim in the presence of a large bony defect. They concluded that arthroscopic stabilization in the presence of such bony deficiencies is likely to fail and requires open surgery. Thus, despite an adequate Bankart repair, consideration must be given toward treating the associated posterolateral defect within the humeral head if it is of sufficient size. Several different reconstructive solutions have been proposed for dealing with large Hill-Sachs lesions. These solutions vary from soft tissue transfers [6] to bony reconstructions such as humeral osteotomy [7], structural osteochondral allografts [8] and transhumeral impaction grafting [9]. Others advocate hemi arthroplasty [10] as a definitive treatment. Recently, Purchase et al [11] presented a technique of capsulotenodesis of the posterior capsule and infraspinatus tendon to fill the Hill-Sachs lesion tendon (also known as the French term "remplissage"). The purpose of our study was to evaluate the outcome of arthroscopic *remplissage *in an unstable shoulder with a large engaging Hill-Sachs lesion. Our hypothesis was that arthroscopic *remplissage *is an effective adjunct to shoulder stabilization in the presence of engaging Hill-Sachs lesions in terms of function and patient satisfaction.

## Materials and methods

Overall, 65 all arthroscopic shoulder stabilizations were performed in our institution from 2007 to 2009. Up to date, 25 patients were identified in whom arthroscopic shoulder stabilization included a capsulotenodesis to fill the humeral head lesion in addition to capsulolabral repair around the glenoid rim. This procedure was done in patients without a significant glenoid bone loss. This study included patients with a minimum follow-up of 2 years (11 of the 25 patients). The diagnosis of recurrent, anterior shoulder instability was made on the basis of a history of recurrent anteroinferior dislocation or subluxation with physical signs of anteroinferior instability. All patients underwent preoperative radiographic and MRI evaluations. The decision to address the lesion was made during arthroscopy if the posterolateral humeral defect engaged the anterior rim of the glenoid in abduction and external rotation of less than 90°, as described Koo et al [12]. Data was retrieved from the surgical reports and follow-up files.

All patients provided formal informed consent for participation in this study.

With a mean follow-up time of 30 months (range 24 to 35) evaluations were performed pre and post operatively by an independent observer according to the shoulder rating scales of Rowe et al [13] and the Simple Shoulder Test (SST) [14]. Table 1 shows patient demographics.

**Table 1:** Demographics

Variable	Data
Gender	All Male

Mean Age (range)	25.5(19.6-38.5)

Mean Follow-Up Time in Months (range)	30(24-35)

Pattern of Instability (Unidirectional, MDI)	(8, 3)

Sports Participation (Professional, Recreational)	(2, 9)

Workers Compensation	0

MDI; multidirectional instability

**Figure 1:**
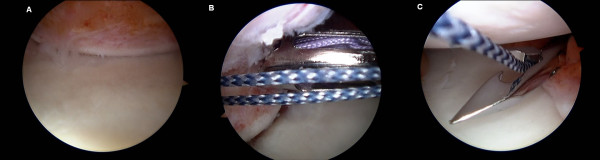
**Images illustrate arthroscopic *remplissage***. **(A) **An engaging Hill-Sachs lesion. **(B) **Anchors are inserted into the humeral head defect. **(C) **A penetrating grasper is used to retrograde the sutures through the adjacent posterior capsule.

All arthroscopies were done by a single surgeon experienced in that procedure. Every operation started with an examination under general anesthesia. Anteroposterior humeral translation was examined with the patient's arm in 90° of abduction and varying degrees of external rotation. The translation was rated as grade 0 (no translation), grade 1+ (translation of less than the margin of the glenoid), grade 2+ (translation beyond the margin of the glenoid with spontaneous reduction), or grade 3+ (translation beyond the glenoid without spontaneous reduction). Inferior translation was measured according to the subacromial sulcus sign. The distance between the inferior margin of the lateral aspect of the acromion and the humeral head was measured and was rated as grade 0 (no sulcus), grade 1 (<1 cm), grade 2 (1 to 2 cm), or grade 3 (>2 cm). In principle, we used a similar surgical technique of the Hill-Sachs *remplissage *already described by Purchase et al [11]. Briefly, the surgery was performed under combined general anesthesia and interscalene block. The patient was placed in the lateral decubitus position on a beanbag support to tilt the trunk approximately 20° posteriorly with the arm in 50° abduction, 20° flexion and 5 kg of traction. Initially a posterior portal was created and then anterosuperior, and anteroinferior portals penetrating the superior and inferior borders of the rotator interval, respectively. The antroinferior portal was the primary working portal for anterior labral repair and the *remplissage *was done through the posterior portal. Diagnostic arthroscopy was performed through the posterior and anterosuperior viewing portals. After evaluating the capsulolabral damage, the arm was temporarily released from traction and the scope was aimed to the Hill-Sachs lesion while dynamic examination in abduction and external rotation was performed under visualization (Figure 1A). If an engaging Hill-Sachs lesion was found in the position of abduction and external rotation of less than 90°, one or two anchors (LUPINE™ BR Anchor w/#2 ORTHOCORD^®^, DePuy Mitec Inc.) were inserted into the Hill-Sachs lesion via the posterior portal. The suture limbs were left untied at that stage (Figure. 1B). The anterior capsulolabral repair was then performed. Finally, the *remplissage *was performed while viewing from the anterosuperior portal. The posterior cannula was withdrawn posterior to the capsule and infraspinatus into the subdeltoid space. A penetrating grasper (Figure 1C) was used to retrograde the sutures through the adjacent posterior capsule. This was done superior and inferior to the initial portal entry site. The sutures were then tied blind in the subdeltoid space.

Post operatively the shoulder was protected in a sling for 4 weeks while performing movements of elbow, wrist and fingers. At week 3 the patient started isometric exercises and at week 4 shoulder external rotation motion. After week 4 the patient was encouraged to perform elevation above 90° and was reviewed by the surgeon and physiotherapist at 6 weeks after the surgery. During weeks 6 to 12 the patient gradually increased elevation and rotation strengthening exercises. Return to sport was allowed after 6 months when at least 90% of shoulder strength and range of motion had been regained.

Results were expressed with descriptive methods (mean, range). The paired Student's t test was used for comparison between scores before and after surgery. P value of less than 0.05 was considered statistically significant.

## Results

There were no recurrent dislocations and no patient had further surgery on his shoulder. At the time of the follow-up all patients had returned to their regular jobs and normal activities including the 2 athletes who had returned to play on professional level. All patients had a large engaging Hill-Sachs lesion which was treated by a *remplissage *utilizing one or two anchors into the humeral head defect. Additional common surgical findings are presented in Table 2. None of the patients had a rotator interval closure.

**Table 2:** Common surgical findings

Variable	Data
EUA	Full ROM, AI translation +3

Labral Defect	Anterior tear, 5 patients had minimal glenoid bone loss (<25% of glenoid width)

Number of Anchors in Anterior Glenoid Rim	3 to 4

EUA; examination under anesthesia, ROM; range of motion, AI; anterior inferior

Overall, the average number of positive responses on the 12-question Simple Shoulder Test were 6.6 before the operation and 11 at the last follow-up (p < 0.001). The Rowe score for instability improved from 10.6 preoperatively to 85 at the last follow-up (p < 0.001) and was considered good to excellent in 78% of the patients (Table 3). While post operative elevation and internal rotation motions were documented as normal, external rotation motion was found to be limited to an average of 83% of the range that was found in the contralateral shoulder. There were no postoperative complications.

**Table 3:** Simple Shoulder Test (SST) and Rowe results

	Total, mean (range)		
**Scores **^a^	**Pre-Op**	**Follow-Up**	**P**

**SST**	6.6 (1-10)	11 (10-12)	<0.001

**Rowe**	10.6 (0-45)	85 (70-95)	<0.001

^*a *^*The maximal points were 12 for SST and 100 for Rowe*

## Discussion

Our findings suggest that performing the *remplissage *technique in conjunction with Bankart repair on unstable shoulders with large engaging Hill-Sachs lesion provides good short term functional results with no recurrent dislocations.

The presence of a large Hill-Sachs lesion can engage with the anterior glenoid rim with the arm in abduction and external rotation levering the humeral head anteriorly. This mechanism has been regarded as a significant cause of recurrent shoulder dislocations and of arthroscopic reconstruction failure [5]. The treatment of osseous defects as part of shoulder stabilization surgery was recently reviewed by Bushnell et al [15] and Lynch et al [16]. Specifically, humeral head defects can be addressed in several ways. The defect can be redirected using the Weber rotational osteotomy [7] to increase the retroversion of the proximal humerus. However, although the published results were good [17] most patients had an internal rotation deficit and there is a considerable risk of malunion or nonunion. Other options are to seal the defect with structural allograft [8] or transhumeral impaction bone grafting [9]. The former requires an extensive open approach with risks of graft or hardware failure while the later is less invasive and more anatomical but might not be suitable for large defects or osteopenic patients. Recently, Chapovsky and Kelly described an all-arthroscopic technique to fill the defect with an osteoarticular allograft [18]. A Prosthetic resurfacing arthroplasty has also been used to treat focal deficits of the humeral head [19,20] but since shoulder instability is mostly encountered in the younger population with a higher likelihood of prosthetic failure it is a less favorable solution. From 2007 the senior author has started to use the *remplissage *technique for instability cases involving a large posterior engaging Hill-Sachs lesion. The decision to perform a *remplissage *was made during arthroscopy. The all arthroscopic technique was previously described by Wolf and colleagues for treatment of combined glenoid loss and a Hill-Sachs lesion [11] and also by Krackhardt et al [6] for a reverse Hill-Sachs lesion. The principle is a fixation of the conjoined infraspinatus tendon and posterior capsule to the abraded surface of the humeral head defect. At the 26th Annual Meeting of the Arthroscopy Association of North America, Wolf et al reported on an unpublished study of 24 patients with a minimum of 2-year follow-up. Twenty two were very satisfied; of these, 15 reported excellent results and 7 had good results. Two patients were rated with poor results. The eight patients in whom prior surgery had failed were without recurrence at follow-up. There were two recurrent dislocations, one due to a motorcycle accident and the other resulting from a wrestling match. They concluded that "Filling the lesion effectively obliterates the Hill-Sachs lesion and converts it into an extra-articular lesion, thereby preventing engagement. There were no significant complications, and the concern that the *remplissage *would limit rotation did not materialize". The patients in our study were mostly a community-based young population but also included two professional athletes (Australian Football players). In our current study the results showed a significant improvement at the last follow-up (mean 30 months) with no recurrent dislocations. We used the Simple Shoulder Test which was previously found reliable, valid and responsive [14] to document functional improvement. Overall, the average number of positive responses on the 12-question Simple Shoulder Test were 6.6 before the operation and 11 at the last follow-up (p < 0.001). We used the Rowe score [13] to assess postoperative stability. The Rowe score for instability improved from 10.6 preoperatively to 85 at the last follow-up (p < 0.001) and was considered good to excellent in 78% of the patients postoperatively.

We share the opinion of Koo et al. [12] on this procedure's advantages. It is a minimally invasive approach to convert an intra-articular lesion into an extra-articular lesion, without the morbidity associated with open procedures and no additional graft material, thereby making the procedure quick and easy to perform.

Even though there is a concern that the tenodesed cuff and capsular tissue can act as a mechanical block to external rotation of the shoulder [21] it was found to be minor in our patients and did not interfere with daily activities.

The strength of this study is in its uniform surgical indication, operative technique, postoperative care and follow-up methodology. Nevertheless it has several limitations. First, all procedures were performed by the same surgeon which might not reflect other people's results. Another drawback is the small number of patients (most participate in recreational sports only) and the relative short follow-up time. This is because the technique was introduced only recently and significant Hill-Sachs lesions are relatively rare. Thus in order to support our results there is a need for long term controlled studies preferably with a larger cohort of patients.

## Conclusions

Arthroscopic *remplissage *for shoulder instability offers an effective soft tissue technique to seal a large engaging Hill-Sachs lesion with respect to recurrence rate, range of motion and shoulder function.

## Competing interests

The authors declare that they have no competing interests.

## Authors' contributions

All authors had substantial contributions in following up the patients and in data collection. DB has performed the surgeries. BH and LM have assisted in surgeries. BH participated in the design of the study and performed the statistical analysis. DB conceived of the study, and participated in its design and coordination. All authors read and approved the final manuscript.

## References

[B1] Yiannakopoulos CK Mataragas E Antonogiannakis E A comparison of the spectrum of intra-articular lesions in acute and chronic anterior shoulder instability Arthroscopy 2007 23 9 985 990 10.1016/j.arthro.2007.05.009 17868838

[B2] Spatschil A Landsiedl F Anderl W Imhoff A Seiler H Vassilev I Klein W Boszotta H Hoffmann F Rupp S Posttraumatic anterior inferior instability of the shoulder: arthroscopic findings and clinical correlations Arch Orthop Trauma Surg 2006 126 4 217 222 10.1007/s00402-005-0006-4 16217670

[B3] Calandra JJ Baker CL Uribe J The incidence of Hill-Sachs lesions in initial anterior shoulder dislocations Arthroscopy 1989 5 4 254 257 10.1016/0749-8063(89)90138-2 2590322

[B4] Palmer I Widen A The bone block method for recurrent dislocation of the shoulder joint J Bone Joint Surg Br 1948 30 53 58 18864946

[B5] Burkhart SS De Beer JFB Traumatic glenohumeral bone defects and their relationship to failure of arthroscopic Bankart repairs: significance of the inverted-pear glenoid and the humeral engaging Hill-Sachs lesion Arthroscopy 2000 16 677 694 10.1053/jars.2000.17715 11027751

[B6] Krackhardt T Schewe B Albrecht D Weise K Arthroscopic fixation of the subscapularis tendon in the reverse Hill-Sachs lesion for traumatic unidirectional posterior dislocation of the shoulder Arthroscopy 2006 22 2 227.e1 227.e6 10.1016/j.arthro.2005.10.004 16458812

[B7] Weber BG Simpson LA Hardegger F Rotational humeral osteotomy for recurrent anterior dislocation of the shoulder associated with a large Hill-Sachs lesion J Bone Joint Surg Am 1984 66 1443 1450 6501339

[B8] Miniaci A Berlet G Recurrent anterior instability following failed surgical repair: Allograft reconstruction of large humeral head defects J Bone Joint Surg Br 2001 83 Suppl 1 19 2011245531

[B9] Kazel MD Sekiya JK Greene JA Bruker CT Percutaneous correction (humeroplasty) of humeral head defects (Hill-Sachs) associated with anterior shoulder instability: a cadaveric study Arthroscopy 2005 12 1473 1478 10.1016/j.arthro.2005.09.004 16376238

[B10] Moros C Ahmad CS Partial humeral head resurfacing and Latarjet coracoid transfer for treatment of recurrent anterior glenohumeral instability Orthopedics 2009 32 8 10.3928/01477447-20090624-21 19708626

[B11] Purchase RJ Wolf EM Hobgood ER Pollock ME Smalley CC Hill-sachs "remplissage": an arthroscopic solution for the engaging hill-sachs lesion Arthroscopy 2008 24 6 723 726 10.1016/j.arthro.2008.03.015 18514117

[B12] Koo SS Burkhart SS Ochoa E Arthroscopic double-pulley remplissage technique for engaging Hill-Sachs lesions in anterior shoulder instability repairs Arthroscopy 2009 25 11 1343 1348 10.1016/j.arthro.2009.06.011 19896057

[B13] Rowe CR Patel D Southmayd WW The Bankart procedure: a long-term end-result study J Bone Joint Surg Am 1978 60 1 16 624747

[B14] Godfrey J Hamman R Lowenstein S Briggs K Kocher M Reliability, validity, and responsiveness of the simple shoulder test: psychometric properties by age and injury type J Shoulder Elbow Surg 2007 16 3 260 267 10.1016/j.jse.2006.07.003 17188906

[B15] Bushnell BD Creighton RA Herring MM Bony instability of the shoulder Arthroscopy 2008 24 9 1061 1073 10.1016/j.arthro.2008.05.015 18760215

[B16] Lynch JR Clinton JM Dewing CB Warme WJ Matsen FA Treatment of osseous defects associated with anterior shoulder instability J Shoulder Elbow Surg 2009 18 2 317 328 10.1016/j.jse.2008.10.013 19218054

[B17] Kronberg M Brostrom LA Rotation osteotomy of the proximal humerus to stabilise the shoulder. Five years' experience J Bone Joint Surg Br 1995 77 924 927 7593108

[B18] Chapovsky F Kelly JDIV Osteochondral allograft transplantation for treatment of glenohumeral instability Arthroscopy 2005 21 1007 16086562 10.1016/j.arthro.2005.04.005

[B19] Scalise JJ Miniaci A Iannotti JP Resurfacing arthroplasty of the humerus: Indications, surgical technique, and clinical results Tech Shoulder Elbow Surg 2007 8 152 160 10.1097/bte.0b013e31806196e6

[B20] Raiss P Aldinger PR Kasten P Rickert M Loew M Humeral head resurfacing for fixed anterior glenohumeral dislocation Int Orthop 2009 33 2 451 456 10.1007/s00264-007-0487-6 18092162 PMC2899085

[B21] Deutsch AA Kroll DG Decreased range of motion following arthroscopic remplissage Orthopedics 2008 31 5 492 19292311 10.3928/01477447-20080501-07

